# Words or code first? Is the legacy document or a code statement the better starting point for complexity-reducing legal automation?

**DOI:** 10.1098/rsta.2023.0160

**Published:** 2024-04-15

**Authors:** Oliver R. Goodenough, Preston J. Carlson

**Affiliations:** ^1^ Vermont Law and Graduate School, South Royalton, VT, USA; ^2^ Thayer School of Engineering, Dartmouth College, Hanover, NH, USA; ^3^ Affiliated Faculty, CodeX, the Stanford Center for Legal Informatics, Stanford, CA, USA; ^4^ CodeX, the Stanford Center for Legal Informatics, Stanford, CA, USA

**Keywords:** legal complexity, computational law, computable contracts, insurance automation

## Abstract

Law is a critical tool that humans have created to assist them in managing complex social interactions. Computational Law holds the potential to significantly enhance our capacity to express and manage legal complexity, and a number of advantages can result from restating public and private legal rules in computable form. Capturing that potential depends in part on the approaches taken to automation. One set of choices involves whether to translate directly into code from existing natural language statements of laws, regulations and contracts or whether to step back, envision the basic structure underlying those statements and build a software approach that reflects that structure in a code-native manner. We argue that many advantages can flow from the second approach, and we present a specific use case of a simplified insurance policy as an example of this approach. Large language models may assist in this process, but are not yet a replacement for a code-native utility.

This article is part of the theme issue ‘A complexity science approach to law and governance’.

## 1. Introduction: law and complexity

Complexity is widely recognized as a property of contemporary legal systems [1–5]. In some ways, however, that puts the cart before the horse. Contemporary human life is complex – with all of us dependent on, and interacting with, many systems and institutions, modes of communication, layers of technology, etc. The function of legal rules is to create some boundaries and institutional structures within the complexity of life as social animals, tools that help human actors manage that complexity and realize opportunities within it. Law may be complex, but, at its best, it helps us to manage the greater complexity of the society that surrounds us.

How does law do this? The core element that distinguishes law from moral suasion is the power of the law to use coercion – incarceration, monetary fines, confiscation and transfer of property – to change the reward/cost structures of an actor's behavioural options. In the language of game theory, this is a mechanism design exercise [6,7]. By adding a layer of legal consequence, the pay-off matrix for interactions can be skewed. In a well-designed legal system, these changes are generally aimed at making predatory conduct, defection and other anti-social options less attractive. This, in turn, creates social space for safety, reliability and cooperation that would otherwise be unavailable. By establishing these pools of safety and reliability, law plays an immensely important role in supporting the relatively benign – and productive – world in which many of us live much of the time. And all of this can be understood as a means of structuring the complexity of human life in a contemporary society so that individual actors can successfully navigate within it.

But as the complexity of economic interactions, technical enablement and other ‘blessings’ of contemporary life has grown, so too has legal complexity, in a feedback loop of management, enabling, development and further management. Technological factors invented over the last few centuries have permitted this explosion of legal and societal complexity. High-speed, inexpensive printing in particular enabled the transition of law from stories and maxims to widely distributed written rules of increasing clarity and elaboration [8]. This increase, in turn, permitted the development of both the modern regulatory state and of a global, interconnected economic system operating on reliable contractual expectations. And with the rise of readable electronic data, legal formulations have been even more widely published by the Internet. This digitization has allowed legal statements to become even more certain, more factually nuanced and more complex, both in themselves and in the behaviours they seek to shape and direct.

But this digital distribution of text-based documents is only a transitional step in incorporating technology into law. The use of PDFs has amplified existing approaches, but has not precipitated great change. By contrast, representing legal rules directly in code – ‘computational law’ – is likely to be revolutionary in its impact [1,9–13].

## 2. Computational Law

The computational law revolution is coming just in time. Written law is now nearly drowning in its own success. In their foundational paper on legal complexity, Ruhl & Katz [2] point to the US tax code as the classic example of an extremely complex law. And this is just an example of a wider phenomenon.

The increase in the complexity of legal rules, however, is not necessarily a bad thing in itself. As noted above, complexity in law, if it is capable of being efficiently accessed and applied, can support successful management of the very complex interaction networks within which humans now live. Take, for instance, the patenting and regulatory approval processes that underpin the development of new drugs. They are complex processes mediated by complex legal rules, and may require years and significant sums to navigate successfully. That said, they have helped to foster the development of nearly miraculous – and mostly safe – treatments for many of humanity's most painful medical afflictions. It is easy to curse those processes when we try to navigate them, but we are probably better off with them than without.

Which is not to say that rule sets such as these cannot be improved. Many of our legal processes and their accompanying rules appear to be nearing the practical boundaries of natural language specification, comprehension and implementation. Even expert lawyers can be challenged trying to navigate the complex webs of event and consequence embedded in regulations and contracts. And relying on humans as the interpreters and implementers is generally a slow process. But this can change. With the development of powerful machine computation, high-speed interconnectivity and cheap digital storage and retrieval, our ability to manage complexity is taking another leap forward: expressing law in executable computer code. The changes this will enable for our legal system are likely to be as profound as the changes that widely available written law enabled in the twentieth century, ushering in the era of the modern regulatory state.

Theorists in legal complexity have recognized the potential of a computational law approach, citing the application of tech, with its digitized data and automated processing, as a way to ameliorate the challenges of that complexity [1,2]. By stating the rule sets in code and then automating the processes of answering queries about the rules sets and their application and execution, the limits of human expertise described above can be transcended in significant ways. And, if the approach to this coding and automation of law is carried out correctly, a number of additional benefits can flow from this. To mention just a few, its structured data and processing, expressed in digital form, can be paired up with user experiences and interfaces that provide much greater transparency and understandability of the rule sets that govern our behaviour (e.g. [14–16]). Queries can be made more effectively; analytical tools can be more easily applied (e.g. [17]). The relatively early step of legal text distribution via the Internet has already increased access to legal rules for many; a fuller move to computational law would help scale this further. Automation can also reduce human error and subjectivity in the application of legal rules, leading to greater predictability and fairness. And in addition to the help such measures would give to individuals interacting with law, cumulatively they would provide a significant increase in the ability of actors – and society as a whole – to manage the complexity of law and, by extension, the complexity of life, in a vastly interconnected, specialized and interdependent world.

Of course, not all of the consequences will be positive. Some of the power of an automated system can be captured and turned against the interests of individuals by an overly intrusive government or by exploitative businesses. While thoughtful legal regimes, such as the European Union's General Data Protection Regulation (GDPR), can help to lessen these negative impacts, they are only as good as the public will and the actual workings of governance permit them to be.

Furthermore, at least some of the gains in complexity management that legal encoding and automation will provide are likely to be eaten up by permitting us to create even more complexity in the law and in the social constructs that law enables. There is the potential for a ‘Red Queen’ dynamic in the cycles of complexity management and creation [18].

Exploring and balancing the potential for benefit and harm from legal automation in its various forms is beyond the scope of this paper, and must be left to others (e.g. [19–21]). But the process appears inevitable, and, on the whole, the potential for good (such as managing complexity) appears to outweigh the concerns. As the process goes forward, there will be more and less productive ways of going about it. In addition to the direct benefits of interpretation and application that can flow from any effective machine-executable version, there is also the potential to improve access, comprehensibility and broader analytics. We believe pursuing approaches to legal automation that realize more of the benefits of translating public and private legal rules into machine-friendly statements of data and code will yield significant social benefit.

In order to meet the goal of realizing the complexity management benefits of legal automation, there are important choices that need to be made about how to go about this automation. The remainder of this paper will explore one such choice set, comparing two basic approaches with the task of representing public and private legal rules in code. The approaches are:

(i) starting with the words of traditional legal specification and seeking, for the most part, to directly automate and encode them into executable form via human programming or via some kind of machine learning approach, such as a large language model – we call this a ‘words first’ approach, or
(ii) stepping back from the current language-based formulations, understanding the abstraction of the behavioural encouragement sought by the legal rules and how that encouragement can be supported by chains of event and consequence, and then creating direct, executable representations of rules supporting such behaviour in the language of code – we call this a ‘code first’ approach.

Put tersely, as we seek to represent the statements of law in computable form, do we start with words or code?

These approaches can apply not only to the transference of existing legal rules and contracts into code; they can also apply to newly created statements. Under the words first approach, the conceptualization of the new statement (whether a law or a contract) would still be done in words, which would then be translated. Under the code first approach, the conceptualization could be realized directly into a code statement, without the natural language intermediate step, although perhaps with the assistance of a creation platform, and with some use of natural language in describing the ontology of events relevant to the law or contract.

The remainder of this paper will focus on this important entry level choice. It argues that in any legal specification that will be referenced or applied with frequency, a code first approach will generally be better. In more one-off, low usage cases, where the cost of abstracting the logic of the legal rule-set and using that understanding as the basis for a re-usable platform is less justified and the pay-offs of easy variability and reuse are less clear, the direct language translation approach may be preferable. The developments of Large Language Model (LLM) capabilities (e.g. [22,23]) can help to support, or even carry out, aspects of both approaches. We will also discuss, if briefly, the possibility of using LLM approaches as a kind of ‘words only’ method, particularly when applied to the corpus of legacy, natural language specifications of rules and bargains.

## 3. Starting with the current words and translating them into code

Some who seek to represent legal rules in code have chosen to take existing, word-based formulations and translate them, pretty much as directly as possible, into a machine executable version (e.g. [24]). The natural language version, frequently seen as a ‘document’ of some kind, is taken as the canonical version of the rule, with automation creating a useful derivative version that allows speedy processing, and opens the door to some further analytics.

Right at the start, there is a fundamental mistake that many make – often as an unexamined assumption – that the legacy document is in fact canonic. We believe that the language representation of a rule or bargain is just that, a representation. It is one way of specifying the abstraction of behaviour and outcome that the law will seek to ensure is imposed. Our somewhat Platonic view holds that there is an essential chain of events and consequences in the world of human social interaction that we are seeking to describe in our legal statements, somewhat like the chain of event and consequence we see at the molecular level in chemistry. The chain exists outside of words, although words are useful tools in seeking to specify the chain. But the chain can be described in pictures [25], or embodied in a physical mechanism, such as a vending machine [7,26], or, as we explore here, in machine executable code.

Because natural language *has been* our best way – in most instances – of making that specification, we have come to think that it is somehow the abstraction itself, rather than just one way of describing it. Using that existing, legacy specification as a starting point for a direct translation exercise may be expedient, even preferable in some cases, but it is not necessary.

The choice to start with the legacy text often overlaps with developing and deploying ‘controlled natural language’ (CNL) approaches to programming. CNL programming typically requires the user to master a set of commands that use a selection of real words, or a stylized syntactical structure resembling speech, or both, to form the rule statement to be represented [27,28]. The CNL system recognizes these statements and compiles them, sometimes with one or more intermediary steps, into machine executable code. The thought is that such stylized prose can be used by ‘domain experts’ (such as lawyers) who are not trained coders to create programs more easily than asking them to master abstracted programming languages such as Java, Python, Solidity, Prolog or C. Some of these translation languages, such as L4, are considered ‘domain-specific languages' (DSLs) for the law [29]. Such languages may have structures closer to those of traditional programming languages than CNLs do, but they are limited to expressing statements about the legal domain. Given that many CNLs are also DSLs, the unclear line between the two, and the similar goals of each in the legal domain, in this paper we consistently use the term ‘CNL’ to refer to both CNLs and DSLs.

CNL systems are not necessary features in a words-first, text translation approach to moving toward computational law, but the two are often linked. The hope is that a moderately skilled CNL practitioner (or even a generative AI system such as an LLM platform) can be turned loose on an existing legal document to produce a usable automated version as a relatively mechanical matter of straightforward restatement. The practitioner reads the text and writes the program as they go through the document, without really having to understand the rules or the transaction that the document is seeking to represent. CNL could also be used for *de novo* drafting by a skilled practitioner, and could even be a tool in the structure/code approach discussed below [9,30,31]. We consider such de novo drafting via a CNL to be an instance of a code-first approach, since languages that can be compiled to machine code must have formally defined semantics. Unless tied to a set of more general standards and structures as part of an integrated model, however, many of the benefits below that can flow from a code-first approach will be hard to attain. A one-off creation will remain just that – useful in its instance, but not enabling more sophisticated, complexity managing, applications.

There are a number of capable CNL approaches that have been applied to law (see the discussion in [32]). While some, such as Logical English [33,34], have more general utility, many have been specifically designed for legal specification. These include L4 [29] and Lexon [35]. L4 seeks to provide a ‘formalism for transcribing rules and reasoning support for verifying their properties', with an emphasis on capturing the defeasible reasoning that characterizes traditional legal text [29]. Logical English (LE) similarly focuses on the expression of rules, but does not emulate traditional legal text to the extent that L4 does, as LE seeks to be a general-purpose CNL for programming. By contrast, Lexon is intended to represent legal contracts as blockchain smart contracts [35]. In doing so, it focuses more on *actions* than on rules/obligations and their analysis, as Lexon is intended to generate code that can execute the terms of a contract [36].

These languages are made machine executable by transpiling them to other languages (in the case of L4) or by embedding them within other programming languages, ranging from declarative languages such as Prolog (LE) to imperative languages such Solidity (Lexon).

Translating existing documents more or less directly into code has attractive features. To begin with, it respects the traditions of legal drafting and of customary business usage, providing comfort to adopters and regulatory or judicial reviewers who do not want to be challenged by a new approach. As a part of this comfort, a ‘doppelganger’ text-based document can often be preserved, and perhaps given precedence over the code translation in the event of a dispute. Provided the translation process creates a relatively accurate code-based version, there is no need for judges – or the legal department in the implementing company – to trouble themselves about understanding something new [1].

Second, the word-first strategy can be a bit easier to deploy, at least as an initial matter. Rather than beginning with stepping back and conceptualizing the law or the bargain in its totality, the conversion process just requires a reasonably skilled practitioner (who may require legal knowledge; see [24]) to make a step-by-step translation of the data and logic in the contract as it currently exists.

Third, such a word-centric approach better enables using a deontic vocabulary in the transition to code, that is, one employing the language of ethical obligation to formulate duties. Concepts like ‘should’ and ‘ought’ have been built into the conversion methodology of some of the legal model languages that start with the word text and then look for a conversion. A discussion of whether this is actually useful in specifying legal formulations as opposed to philosophical statements is beyond the scope of this paper.

Finally, natural language processing may be able to assist translation in a very direct way. Using the code-writing abilities of LLMs, this approach lets the AI act in place of that reasonably skilled practitioner to undertake the conversion, at least as an initial matter. While there are currently accuracy concerns in relying too fully on an LLM-produced outcome, such a process could create a time-saving first draft in code that could be de-bugged and corrected in a later review [37].

## 4. AI application without translation

Some have concluded that LLMs and other Natural Language Processing (NLP) approaches can go a big step further. Rather than simply helping a translation to code, LLMs can make it possible to use the existing natural language text as the computational artefact in itself, without any representation in a traditional machine-executable language. A query about application or interpretation of legal text can be posed directly to the machine, which will deduce an answer directly from that text. This approach has some promise, particularly in contexts such as summarization or interfaces for questions about outcomes in a particular factual context. LLM-based consumer facing education and advice utilities on legal subjects are being rushed in service in a number of contexts (e.g. [21,38]).

While current LLM systems can locate key provisions in a contract or other legal text, and even, to some degree, follow and draw conclusions from the logic imbedded in those texts, reliability at this stage is questionable enough that human review and curation is a necessary step in contexts where granular accuracy is critical. In a widely publicized case in June 2023, lawyers in the United States found themselves in trouble for submitting a ChatGPT authored brief in a federal court in New York, only to discover that the AI service had completely fabricated case precedents and citations [39]. The current versions of this platform are optimized for coherence, and not for accuracy. Human review remains critical (e.g. [40]).

Access to analytical tools beyond text generation is important for legal complexity management, and LLMs can support data analytics projects. This is a rapidly emerging field, and a comprehensive treatment is not within the scope of this paper (see [23]). In itself, the ability to make specific queries about a natural language legal text does not enable more complex analytical processes of the kind now practised in sophisticated ‘big data’ techniques (e.g. [40]). But a number of techniques, such as sophisticated training sets (e.g. [41]) and prompt engineering (e.g. [42,43]), are permitting LLMs to be part of more powerful and comprehensive analytics applied to larger bodies of text.

Techniques such as these may already make LLM approaches useful tools in dealing with the large and important corpus of existing legacy agreements and laws expressed in natural language [44]. That said, the application of big data style analytical tools to the more structured set of data and programs that arise when one directly represents the legal statements in digital form is well established (e.g. [17]). At least in the short term, a direct reliance on AI for automating legal statements may not match the outcomes possible with a rules-based system developed specifically to be represented in code.

Code-first and LLM approaches need not stand in opposition to each other. LLMs may be useful in an intermediary step of information extraction, pulling particular data points from natural language statements [40]. It may also be possible to use LLMs to assist in creating elements for use in a more structured legal automation process. Such techniques could help make a code-first transition more scalable, saving time and cost. We anticipate that as the capacity of LLMs continues to evolve, the best outcomes may come from a combination of LLM and code-first approaches [44].

## 5. Expressing the rules and their structure in a code-native representation

Treating the words of legacy, natural-language documents as the canonical statement of legal rules – whether in laws or contracts – is not the only path forward. Rather, we can also consider how best to represent the understanding about behaviours and outcomes that make up laws and bargains directly and natively in code.

A number of benefits will flow from this. First, it will enable embodying the rule or the bargain in well-structured, unambiguous programming code, rather than tracking the convoluted statements that arise in legalistic prose as humans seek to use natural language as a computational statement medium [45].

Second, it will enable deploying interfaces that can liberate the user experience from the shackles of ponderous, opaque legal language, instead enabling graphical techniques and other design elements to communicate the terms (e.g. [46]). The improvement of Windows on DOS provides a useful historical analogy.

Third, the structured data that such platforms naturally create and consume will provide much enhanced opportunities for analysis and guidance – for governments, companies and consumers. These analytics have the potential to be of significant use in complexity management. While turning AI loose on unstructured data can produce useful results, turning it loose on data that are already in easily accessed, machine-native formats increases the power and speed of the exercise considerably.

And fourth, it could allow – and encourage – the designers of laws, regulations and bargains to develop new approaches and formalisms that might have been hard to conceptualize using words but might be more easily envisioned in a code-first environment. By way of a partial example, some of the insights about the difference between the expected ‘happy path’ and the default paths in the loan agreement described by Flood & Goodenough [47] only emerged when they developed the graphical representation of the agreement as a deterministic finite automaton.

The hard step is that the code-first approach needs a transaction structure domain expert, such as a lawyer, to conceptualize abstractly the structure of the legal rules or of the bargain that is to be specified (although even translation-based approaches benefit from such an expert - [24]). With this conceptualization as a guide, we can then create a platform that (i) allows this structure to be represented efficiently and accurately in code; (ii) allows variation in the design of the specific elements of a particular family of rules or bargains to be easily inserted or removed, probably from a somewhat general template for that family; and (iii) provides interfaces for the various users of such a platform that allow them relatively easily to design variations and to submit different types of questions about data and outcomes. Such an interface should allow non-coders to take control of specifying rules. CNL programming approaches have a similar goal, but realize it through awkwardly stylized language applications. Because it is grounded in a pre-linguistic vision of the rule or bargain, a code-first platform can more easily deploy a mixture of non-linguistic interactive features such as toggles, drag and drop, and mouse-over menus, which can enable more precise interaction with the rule or bargain without the need to understand legal language.

What does this process look like in practice? An illustrative use case involves representing an insurance bargain through a computable contracting platform built on this code-first approach.

## 6. Representing an insurance bargain through a code-first approach

Insurance contracts are particularly suited to a code-first approach. The insurance bargain is a logical abstraction whose propositions are linked to events and states in the physical world, plus some arithmetic about benefits and some process roadmaps. We have traditionally represented those elements in words, but that representation is a secondary level abstraction – not the essential chain of events and consequences that are the bargain itself. Moving to code gives us the chance to create a new secondary level description that maximizes the potential of an automated system. This exercise is informed by prior word-based practice, but does not translate it directly.

Getting behind the words, insurance bargains have a recurring structure that shapes their terms. The details within those terms may vary, and may have considerable complexity, but that variation and complexity generally fit within this recurring structure. The elements of this structure are:

1. Is the policy in effect at the time of the events that give rise to a claim? By way of example, is the policy contract signed? Has the premium been paid? Has the term of the coverage expired?
2. Have all conditions necessary to keep coverage in effect been met? Has a required periodic fire safety inspection occurred? Or a required doctor visit?
3. Has a claim proving the occurrence of the required elements for a valid indemnity claim been made? The provisions specifying this are sometimes called the indemnity clause. The contract may lay out different pathways for what constitutes such a valid claim.
4. Is the claim disallowed because of the existence of an applicable exclusion? Was the accident caused by a prohibited activity, such as skydiving, or by driving while intoxicated? Was the business interruption caused by a pandemic?
5. If a valid claim has been made, what is the amount of any payment due, applying the arithmetic of the policy. Are there deductibles? Co-pays? Maximum pay-outs?
6. Ancillary matters relating to process, administration and disputes. What law applies? For a dispute, courts or arbitration? What is the time frame for steps in the claims process? Where are notices and claims sent? What documentation will be necessary?

These elements are sometimes stated in complex ways – poor drafting of traditional policies sometimes interleaves these concepts. Exclusions may appear either separately and be identified as such, or they may be inserted as limits in the coverage provisions themselves. Coverage terms can have exceptions, and those can themselves have sub-exceptions. And, particularly in individually negotiated commercial policies, the various provisions can be stated not just in the body of the core policy, but also in riders, amendments and endorsements that supplement and alter that core policy. In some markets, these policy variables are called the ‘wordings’ – and many companies have standardized libraries of natural language clauses that can be deployed (e.g. policy wordings available at [48]).

But underlying this apparent complexity, in most instances, are the bargain elements described above. No matter how convoluted the policy, the question of whether or not coverage is triggered effectively boils down to a long logic statement of whether specified facts and states about the world are true or false. If the necessary trigger elements for a claim (accident, sickness, casualty damage, etc.) are present and the disabling trigger elements for exclusion or termination of the policy (drunkenness, lack of care, failure to pay a premium, etc.) are not present, then a valid claim has been made. The amount of payment under the claim then depends on a further set of facts and some often-complex arithmetic (deductibles, caps, co-pays, etc.). But here, too, the underlying formula is an abstraction capable of representation in many modes – including code.

Using these elements as a starting point, an effective, easily adapted and widely deployable platform for representing insurance bargains directly into code can be created. While the words of existing policies will likely be mined to help with the ontologies of events and states that will be the elements queried to decide whether coverage is triggered and for how much, the queries themselves can be built directly into code. Specific legacy policies will still be useful references in figuring out coverage methods and targets, but they will stand as a resource, and not as the object of line-by-line translation.

This recurring general structure can inform how we program the data and the queries on that data that together make up a policy. With some additional granularity, the ‘local logic’ of a particular family of policy variations – life, casualty, cyber, workplace – can be realized as a starting point for a template within such a platform [14]. In such a case, the platform will also provide users with the ability to make both standard and bespoke variations within the boundaries of such a family and of such a template. As we will explore below, these code-first representations may be able to enable greater extensibility, analytical capacity and efficiency than a dogged policy-by-policy direct automation of the words could accomplish.

Logic programming languages, such as Prolog or Epilog, will be particularly effective for representing these elements, and, just as importantly, in enabling comparisons, gap analysis, portfolio reviews and other critical complexity-managing by-products of the automation process [44,49]. While a representation of a contract in logic programming can also be achieved through a direct text conversion approach, we believe that such an approach will, in the end, suffer from the limitations on analytical capacity described above unless they are integrated into a bargain-centric platform of the kind at the heart of the more fundamental code-first approach.

## 7. Illustration of concept – automating insurance contracts

A direct demonstration of such a platform, at a simple, illustration-of-concept level, can help explain this approach. Our use case grows out of research we have undertaken as part of the Insurance Initiative at CodeX, the Stanford Center for Legal Informatics. The starting point is an abbreviated contract for a ‘hospital cash’ policy, set out as appendix Illustration 1 below. The point of the policy is to provide a set payment – without worrying about demonstrating actual costs – for any qualifying days of a qualifying hospital stay, up to a set limit. The core policy terms require signatures and payment, a valid claim to be made and certain exclusions not to be true. The version we have developed is simplified, but still has a few ‘moving parts’ that can be varied to create different coverage terms. These include the top permissible age, the location of the hospital and the presence of certain occupational exclusions. This short policy has been developed based on actual commercial models, and the bargain it represents has validity as a research target.

The structure of the policy fits squarely with the core bargain elements for insurance generally described above. And, following the general logic of those elements, its bargain on coverage can also be represented as a specific formula in propositional logic:

((is signed ∧ paid premium) ∧ (condition met ∧ cancelled)) ∧ (claim made ∧ stayed overnight ∧ resulted from sickness or injury ∧ us hospital ∧ stay during policy period) ∧ (skydiving ∨ military ∨ firefighter ∨ police ∨ 65 or older ∨ 75 or older).

Each propositional symbol corresponds to a Boolean condition that may be met by the state of the world (e.g. the policy may have been signed by an insuree, the insuree may be 65 years of age or older, or their hospitalization may have been caused due to a skydiving incident). Symbols grouped by parentheses correspond to conditions that are grouped together in the underlying policy (e.g. the final set of conditions correspond to the policy's general exclusions). Such a propositional formula could instead be structured in conjunctive or disjunctive normal form, but at the cost of the similarity in logical structure between the formula and the contract conditions. The values of the propositional symbols describe the state of an insurance claim and policy, and the overall formula is satisfied if-and-only-if the claim should be covered by the insurance policy.

This formula thus captures the coverage terms of the contract without the use of natural language. Of course, natural language is still present in the definitions of the events and states whose truth or falsity are encoded in the propositions. We also note that this formula leaves out the arithmetic and the process elements, but fully captures the core coverage terms. We have taken the next step in the process and have constructed a simple application that can represent such a policy, with the possibility of easy variation of terms and application of analytics – in this case answering the basic questions of whether a submitted claim is valid or not, and for how much if so. As we proceeded, we did not ignore our word-based representation of the contract; we mined it for an ontology of events and states for use in our code-first approach. The product of the ontology exercise is set out in appendix Illustration 2. We also mined it to help us fill in how the bargain underlying the word-based version fits to the basic structures of insurance. The natural language version is an important resource. What we did not do is try to translate it directly into code. The code-based representation was built using the Epilog logic programming approach from the bottom up based on the recurring structures of insurance.

Let us describe the process we followed. As a first step, we encoded the underlying bargain as a logic program. To do so, as previously stated, we did mine the word-based model for its ontology. But we took those salient points of event and status and built them into an abstracted representation of the bargain using the language of relational logic. The lawyer in our team took the lead as the domain expert in the extraction process, and made a simple list of the necessary elements of the ontology for this kind of contract.^1^ Information relevant to determining coverage (whether about the world, insurees or claims) became the input (or ‘base’) relations of the program. The logic of the policy that determines whether coverage is triggered for specific claims was then encoded as the output (or ‘view’) relations of the program. These output relations (such as ‘policy in effect’, ‘exclusion met’ and ‘coverage present’) were defined in terms of the input relations. While we did not employ any AI assistance in this process, we anticipate that LLMs may be able to undertake some of this task – with appropriate human curation. We plan to explore this in future research.

As a second step, we specified many discrete changes that could be made to the terms and conditions of the policy that, when made, would correspond to variations on the Hospital Cash Policy (e.g. with different exclusions, payout or requirements on hospitalization location). Due to the extensibility of logic programs and our focus on a single ‘policy family’, any of the specified changes could be made in conjunction with the others to yield a policy variation with minimal additional work necessary to make them interoperable. This extensibility of logic programs is due to their declarative and side effect-free nature. As a baseline, a complex logical definition may be added to a set of logical rules with no unintended behaviour, as long as the input relations are known. Achieving similar guarantees with an imperative program usually requires (i) that the program be architected explicitly with extensibility in mind; (ii) that the person writing additional logic is aware of the existing architecture; and (iii) that authors of both the existing and additional logic refrain from writing the side-effectful code that most imperative languages are designed to express.

Third, to demonstrate how such an approach can power an accessible user experience, we created a basic interface that allows the variable elements to be toggled on and off by a non-coder. And because we recognize the utility for legacy comprehensibility, we incorporated into the display of this interface a natural language representation of the policy contract – constructed from the same underlying representation as the code. The UI took the form of three side-by-side panels, presenting (i) the interface for specifying changes; (ii) the natural language version of the policy; and (iii) the corresponding Epilog code. See the screenshot in appendix Illustration 3.

Finally, this representation of the policy contract can be applied to core questions about its operations, and we paired the policy construction screen with a claim evaluation screen. This asks the customer to enter information that gives truth values to the elements of the ontology that matter to the bargain (i.e. defining the input relations). Once these are in place, queries based on the policy as formed can quickly be applied to a claim that has been made to determine whether it is valid and, if not, to identify the elements that are lacking. This in turn can inform both the customer and the insurer about next steps of claim denial or benefit calculation and processing. See the screenshot in appendix Illustration 4.

The underlying code for this representation is available at https://github.com/codexstanford/formation-demo.

While we have not yet integrated further capabilities on top of these, many additional functionalities can be envisioned. On the customer side, coverage options can be compared and contrasted, and gaps and overlaps identified. This kind of ‘portfolio analysis’ can help customers avoid both over- and under-insurance decisions [50]. On the company side, making the claims administration process more efficient would be a significant benefit. While estimates vary, all agree that such costs are a large portion of an insurance company's expenses, and automation can reduce this significantly (e.g. [51,52]). But this is just the starting point. Our approach also permits queries within a company's policy portfolio about exposure to particular risks to be quickly and accurately made [53]. At the start of the COVID-19 pandemic there was a frenzied scramble by insurers to evaluate exposure under their business interruption policies, with teams of lawyers combing pdfs and paper files to evaluate just what had been promised and what was excluded. A system such as ours could have reduced this scramble to a deliberate set of IT activities involving automated queries of the database/ontology and the library of policy forms to see (i) whether there were events specified relating to a pandemic or similar terms; and (ii) what query sets making up policies made reference to those events and with what effect.

A well architected logic programming approach with a systemic focus would enable such querying; analytical requests such as these would be harder – or perhaps impossible – to achieve with an automation process based on a words-first approach, whether through a document-by-document translation or through an AI parsing of existing documents.

Because programming languages have formally-defined semantics, using code as the canonical form of the insurance bargain would ensure that the insurance bargain would be similarly well defined. This would then enable the use of tools for *static code analysis*. Such tools have been developed to ensure programs behave as intended and obey design rules, e.g. Microsoft's StaticDV for analysing drivers for ‘defects and design issues' [54]. Applied to a code-first contract, such techniques could be used to guarantee that the bargain expresses the intended coverage terms, and to understand the coverage it expresses in extreme circumstances, such as the aforementioned pandemic-induced business interruption.

The code-first approach, while using the existing documents as an information resource, can result in well-planned platforms that represent the legal formulation in ways that exploit the advantages of programming, rather than prioritizing a faithful replication of a word-based model. As we have explored, code-first approaches to legal automation can result in structured data, extensibility, ease of querying and an openness to techniques of formal static analysis. While a well-planned words-first approach might enable some of this as well, these capacities are native to the code-first methodology we have described in this paper. And it is techniques like these, on top of simple automation of the legal formula itself, that have the potential to increase significantly the capacity of human actors, individually and coming together as enterprises and government, to use law to manage the complexity of life.

## 8. Implications and next steps

The illustration of the Hospital Cash Policy discussed above demonstrates the viability of a code-first approach, and shows some of the useful flexibility, extensibility and data analytics that can flow from taking this starting point. In our own research, we are seeking to apply the approach to different insurance contract families, longer policies and policies with more complex layers of coverage and exclusion. And while our illustration involves a particular type of bargain, we believe the approach has validity for many other classes of transactions and for public laws and regulations.

On the transaction side, the logic programming approach seems best suited for ‘trigger’ or ‘boundary’ contracts such as insurance or a Non-Disclosure Agreement (an ‘NDA’) [49]. The key questions in such agreements boil down to whether the necessary elements for triggering an affirmative outcome are present or whether some violation of the set of boundary conditions (like a forbidden disclosure under an NDA) has occurred. Some contracts involve a more elaborate branching structure of possible obligations and outcomes, such as the classic happy path/default path structure of many financial contracts (e.g. [47]). Programming such agreements may be best accomplished via a mix of imperative and declarative approaches (e.g. [55]).

Nor is the approach limited to the private sphere of contracts. In ‘The British Nationality Act as a Logic Program’, the classic paper in the legal automation field [9], the authors demonstrate that this particular law also follows the structure of a logic program; many other laws appear open to a similar classification. Logic programming-driven applications that encode municipal building codes have already been deployed [56,57].

There is an important place for LLM-style AI in the code-first approach. As mentioned above, the development of the ontologies, and even of the logic structure itself, are promising tasks where LLMs can be very helpful. But the goal is to build a more direct system that will provide the core computational approach, rather than turning the brute force of pattern-recognizing AI loose each time there is a query for the law or contract.

Representing existing, relatively infrequently queried laws in code may be the best use case for the words-first translation method. While we can envision a code-first legislative platform where assembling legal logic strings can be done natively on a drag-and drop basis, the resulting representation of any existing law would probably require a re-enactment by the legislature of the new platform-based version, although some kind of omnibus action could be taken if there was the appetite. New legislative and regulatory drafting is a better target for the code-first approach; developing a programming utility for constructing laws this way from the ground up is a development that can happen as soon as resources are devoted to it. We anticipate that LLM techniques can help populate such a utility, making the process easier to apply at scale.

## 9. Conclusion

Law is a critical tool that humans have created to assist them in managing complex social interactions. Much of modern life has been made possible by the presence of reasonably reliable legal regimes that protect against predation and enable collaboration. But as the complexity of society has increased, so has the complexity of law, to a point where we are pushing the effective limits of traditional systems of word-based legal rules. Computational Law holds the potential to significantly enhance our capacity to express and manage legal complexity, and a number of advantages can result from restating public and private legal rules in computable form.

How effectively we can capture that potential depends in part on the approaches taken to the automation process. We argue that one set of choices about legal automation involves whether to translate directly into code from existing natural language statements of laws, regulations and contracts or whether to step back, envision the basic structure underlying those statements and build a software approach that reflects that structure in a code-native manner.

We have argued that many advantages can flow from the second approach, and we have presented an example of how we have gone about executing it in the specific use case of a simplified insurance policy. Although there may be some initial costs that must be borne in following this approach, in cases where there are likely to be reasonable volume in use and reasonably high liberty or economic issues are at stake, these costs can be quickly recouped. Private insurance contracts meet those criteria, and our example involves how to automate insurance bargains.

There are cases where the words-first approach, either through translation or the direct application of LLM style artificial intelligence, may be preferable as a matter of dealing with legacy documentation. Going forward, however, we believe that a code-first approach, intelligently developed and deployed, holds the greater promise for allowing society to grasp the many benefits – including complexity management – that can flow from effective legal automation.

## Data Availability

The underlying code for the application discussed in the paper is available at https://github.com/codexstanford/formation-demo.

## Footnotes

1 It might be argued that this process is simply a different version of ‘words first’. As discussed elsewhere, we draw a distinction, however, between (i) using existing word-based formulations as a resource as we construct a code-native representation of the bargain or other legal rule-set; and (ii) trying to translate the word-native representation into code. It is this distinction that we are seeking to capture in our terms ‘words first’ and ‘code first’.

## Appendix A

**ILLUSTRATION 1: SUPPLEMENTAL HOSPITALIZATON CASH POLICY**

Between:

**CODEX INSURANCE LIMITED (us)**

and

______________________________________(you)

This policy is provided on the following terms and conditions:

**1. POLICY IN EFFECT AND CONDITIONS**

1.1 The payment of any benefit under this policy is conditioned on the policy being in effect at the time of the hospitalization for sickness or accidental injury on which the claim for such benefit is premised. The policy will be in effect if:

(a) This agreement is signed,

(b) The applicable premium for the policy period has been paid, and

(c) The condition set out in §1.3 is still pending or has been satisfied in a timely fashion and

(d) The policy has not been cancelled.

1.2 Cancellation will be deemed to have occurred if there is fraud, or any misrepresentation or material withholding of in any information provided by you to the Company in connection with any communication or information relating to this policy, or if the condition set out in §1.3 has not been satisfied in a timely fashion. It will also be automatically cancelled at midnight, US Eastern time then in effect, on the last day of the policy term described in §5 below.

1.3 No later than the 7th month anniversary of the effective date of this policy, you will supply us with written confirmation from the medical provider in question of a wellness visit for yourself with a qualified medical provider occurring no later than the 6th month anniversary of the effective date of this policy.

**2. BENEFITS**

2.1 If you have been confined in a hospital as a result of sickness or accidental Injury, we will pay you the Daily Hospital Income Benefit shown in §5 below.

2.2 The Daily Hospital Income Benefit will only be payable for each (24 hour) day of continuous confinement in a hospital in the United States, from the first day of confinement and for a period not exceeding three hundred and sixty-five (365) days for all such confinement due to sickness or accidental Injury.

2.3 To trigger any benefit, a claim must be made to the Company setting out the basis for making the claim and for there being no exclusion or cancellation.

**3 GENERAL EXCLUSIONS**

3.1 Your policy will not apply to, and no benefit will be paid with respect to, any event causing sickness or accidental injury arising directly or indirectly out of:

(a) Skydiving; or

(b) Service in the military; or

(c) Service as a fire fighter; or

(d) Service in the police; or

(e) If your age at the time of the hospitalization is equal to or greater than 75 years of age

**4. GENERAL CONDITIONS**

4.1 Where does Your Policy apply?

4.1.1 Your Policy insures You twenty-four (24) hours a day anywhere in the world.

4.2 Arbitration

4.2.1 If any dispute or disagreement arises regarding any matter pertaining to or concerning this Policy, the dispute or disagreement must be referred to arbitration in accordance with the provisions of the Arbitration Act (Cap. 10) and any statutory modification or re-enactment thereof then in force, such arbitration to be commenced within three (3) months from the day such parties are unable to settle the dispute or difference. If You fail to commence arbitration in accordance with this clause, it is agreed that any cause of action and any right to make a claim that You have or may have against Us shall be extinguished completely. Where there is a dispute or disagreement, the issuance of a valid arbitration award shall also be a condition precedent to our liability under this Policy.

In no case shall You seek to recover on this Policy before the expiration of sixty (60) days after written proof of claim has been submitted to Us in accordance with the provisions of this Policy.

4.3 Laws of New York

4.3.1 Your Policy is governed by the laws of New York.

4.4 US Currency

4.4.1 All payments by You to Us and by Us to You or someone else under your policy must be in Unitied States currency.

4.5 Premium

4.5.1 The premium described in §5 below shall be paid in one lump sum at the signing of the policy.

4.6 Policy Term

The term of this policy will begin on the date accepted by Us as signified by our signature of the policy (the effective date) and will last for a period of one year from that date, unless previously cancelled pursuant to §1 above.

**5. BENEFIT AND PREMIUM AMOUNTS**

5.1 The Daily Hospital Benefit amount under this policy is $500.

5.2 The premium amount for the policy is $2000.

**6. SIGNATURE**

Please indicate your agreement by signing on the line designated below and returning this to us with your check for the premium.

______________________________ DATE: ____________________

OUR SIGNATURE: _______________________________ DATE: _____________________

**ILLUSTRATION 2**

**Event Ontology for Simplified Hospital Coverage**

A. Policy in effect and condition events
  1. Agreement Signed
  2. Applicable premium for the policy period paid
  3. Policy not cancelled
    a. Fraud
    b. Misrepresentation
    c. Material withholding of information
    d. Midnight, last day of the policy period
  4. Written confirmation no later than the 7th month anniversary of the effective date from a medical provider of a wellness visit having taken place no later than the 6th month anniversary of the effective date
B. Claim element events
  1. In a hospital
    a. Location: in the United States
  2. Result of sickness or accident
  3. Days of continuous confinement (numerical value)
  4. Claim made
C. Exclusion elements
  1. The event causing the sickness or accidental injury arose directly or indirectly out of:
    a. Skydiving
    b. Service in the military
    c. Service as a fire fighter
    d. Service in the Police
  2. Your age is over:
    a. 65
    b. 7
    c. 5
D. Amounts
  1. Daily Benefit $
  2. Premium $
  3. Co-pay $
  4. Deductibles $
  5. Cap $
E. General Terms
  1. Policy term (months)
  2. Other terms omitted (Ricardian on choice of law, arbitration, etc.

**ILLUSTRATION 3**

**ILLUSTRATION 4**
